# 

**Published:** 1889-08

**Authors:** 


### TABLE OF CONTENTS.

ORIGINAL AND SELECTED.

Sanitation, by J. P. Stephens, M D. .... .... 281
Dentition, by Walter A Crow, B. S., M. D.. .... 286
Antifebrin in the treatment of epilepsy .... .... 291
Treatment of gall-stone, &c. .... .... ...... 293
Rigors .... .... .... 294

ABSTRACTS &c.

Prevention of bedsores; Morbus basedowii cured by pregnancy; What are the thoughts of the Dying? .... .... 298
The morphine habit; The etiology of exophthalmic goitre; Mumps; A new source of morphine .... .... 299
Ural; Danger of giving chloroform by gas light; What dressing shall lie next the wound? .... 300
Astringent washes; Sugar in the urine; Abortion .... .... 301
Morphine as an anti-emetic; Action of antipyrin on the teeth; Cocaine in sea-sickness; The period of incubation in eruptive fevers .... 302
Neutralizing cobra poison; Chinese anæsthetic; Myrtol .... .... 303
Ice in the sick room; Salt in milk for children; Salicylic acid .... .... 304
The American Medical Association .... .... 305
A combination of narcotics and exhilarants; The prevention of conception; Use of rhus aromaticus in incontinence of urine .... .... 307
Rectal troubles; A new use for ether during anæsthesia .... .... 308

SCIENTIFIC.

The electrical omnibus in London; Brains in business; Counting the motes .... .... 309
Whence the strength; Nicotine; cause of intermittent fever; Cryolite; The sources of beautiful Colors .... .... 310

NOTES AND FORMULÆ.

Treatment of granular lids; Anodyne liniment; Local counter irritant for children; Catarrhal diarrhœa of children .... .... 311
Antifermenting powder; Antiseptic mixture... 312

EDITORIAL.

Gastrostomy for occlusion of Œsophagus .... 313
What next .... .... 344
Principles and practice of dentistry; A system of obstetrics .... .... 315
Psychology as a natural science; The American Rhinological Association .... .... 316
Synopsis of human anatomy; Convention; Attention; Digestive ferments in intestinal disorders of infants .... .... 317
Baltimore College of Physicians and Surgeons. 318
Special notes .... .... 318, 319, 320

